# Hybrid genome assembly of *Shigella sonnei* reveals the novel finding of chromosomal integration of an IncFII plasmid carrying a *mphA* gene

**DOI:** 10.1099/acmi.0.000189

**Published:** 2020-12-09

**Authors:** Dhiviya Prabaa Muthuirulandi Sethuvel, Shalini Anandan, Dhivya Murugan, Kalaiarasi Asokan, Karthick Vasudevan, Jobin John Jacob, Kamini Walia, Joy Sarojini Michael, Balaji Veeraraghavan

**Affiliations:** ^1^​ Department of Clinical Microbiology, Christian Medical College, Vellore 632004, India; ^2^​ Division of Epidemiology and Communicable Diseases, Indian Council of Medical Research, New Delhi 110029, India

**Keywords:** azithromycin, *ermB*, hybrid assembly, IncFII plasmid, *mphA*, *S. sonnei*

## Abstract

Azithromycin is increasingly being used for the treatment of shigellosis despite a lack of interpretative guidelines and with limited clinical evidence. The present study determined azithromycin susceptibility and correlated this with macrolide-resistance genes in *Shigella* spp. isolated from stool specimens in Vellore, India. The susceptibility of 332 *Shigella* isolates to azithromycin was determined using the disc diffusion method. Of these, 31 isolates were found to be azithromycin resistant. The azithromycin minimum inhibitory concentration (MIC) was determined using the broth microdilution method. In addition, isolates were screened for *mphA* and *ermB* genes using conventional PCR. Furthermore, an isolate that was positive for resistance genes was subjected to complete genome analysis, and was analysed for mobile genetic elements. The azithromycin MIC for the 31 resistant *Shigella* isolates ranged between 2 and 16 mg l^−1^. PCR results showed that a single isolate of *Shigella sonnei* carried a *mphA* gene. Complete genome analysis revealed integration of an IncFII plasmid into the chromosome of *S. sonnei *, which was also found to carry the following resistance genes: *sul*1, *bla*_DHA1_, *qnr*B4, *mph*A, *tetR*. Mutations in the quinolone-resistance-determining region (QRDR) were also observed. Additionally, prophages, insertion sequences and integrons were identified. The novel finding of IncFII plasmid integration into the chromosome of *S. sonnei* highlights the potential risk of *Shigella* spp. becoming resistance to azithromycin in the future. These suggests that it is imperative to monitor *Shigella* susceptibility and to study the resistance mechanism of *Shigella* to azithromycin considering the limited treatment choices for shigellosis.

## Introduction

*Shigella* species are a common cause of dysentery among children, mainly in low- and middle-income countries. Treatment of *Shigella* infection with antibiotics is usually recommended to reduce the complications and risk of transmission [1]. However, antimicrobial resistance (AMR) in *Shigella* spp. is not uncommon. Recently, increasing resistance to ciprofloxacin made third-generation cephalosporins and macrolides the drugs of choice. However, the current emergence of extended-spectrum β-lactamase-producing strains means that azithromycin is now the drug of choice, particularly for children [1–3].

Macrolides are being widely used for enteric pathogens, as they can achieve high intracellular concentrations and produce adequate levels in the colon [4, 5]. Notably, worldwide, azithromycin is being used successfully for shigellosis, despite no clinical breakpoints being available either from the Clinical and Laboratory Standards Institute (CLSI) or the European Committee on Antimicrobial Susceptibility Testing (EUCAST). CLSI guidelines only suggest epidemiological cut-off values for *Shigella flexneri* and *Shigella sonnei *. Also, clinical evidence for the efficacy of azithromycin is limited [2].

Macrolide resistance involves a variety of mechanisms, most of which have already been reported in *Enterobacteriaceae *. These include: (i) target site modification by methylases encoded by *erm* genes, mainly *ermA*, *ermB* and *ermC*; (ii) inactivation by modifying enzymes such as esterases or phosphotransferases encoded by *ereA*/*ereB* or *mphA*/*mphB*/*mphD* genes, respectively; (iii) acquisition of efflux pumps, *mefA* and *msrA* genes have been identified in Gram-negative organisms, though they are found essentially in Gram-positive organisms [5]. Azithromycin resistance in enteric pathogens is commonly due to two mechanisms: drug inactivation by the *mphA* gene, and target-site modification by the *ermB* gene [6]. These genes are usually carried by different incompatibility (Inc) plasmids. The most commonly reported plasmids in *Shigella* are the IncFII type [7]. The objective of the present study was to determine azithromycin susceptibility and to correlate this with the presence of macrolide-resistance genes in *Shigella* spp. isolated from stool specimens in India.

## Methods

### Bacterial isolates and susceptibility testing

A total of 332 *Shigella* spp. were obtained from stool specimens collected between the years 2017 and 2019 at Christian Medical College, Vellore, India. Genus and species identification were performed using conventional methods [8]. Serogrouping of *Shigella* spp. was done by slide agglutination using commercial antisera (Denka Seiken). Antimicrobial-susceptibility testing of isolates against ampicillin, trimethoprim/sulfamethoxazole, ciprofloxacin, cefotaxime, cefixime and azithromycin was performed by Kirby–Bauer disc diffusion method. The azithromycin minimum inhibitory concentration (MIC) was determined by broth microdilution for the isolates that were resistant using the disc diffusion method. The results were interpreted using CLSI 2017 guidelines [9].

### Analysis of *mphA* and *ermB* genes by PCR

Total DNA was extracted using a QIAamp DNA mini kit (Qiagen). The isolates were analysed for the presence of *mphA* and *ermB* genes by PCR, as described elsewhere [5]. Known positive and negative controls were used in every run.

### Whole-genome sequencing

The *Shigella* isolate positive for a macrolide-resistant gene was further subjected to whole-genome sequencing. Genomic DNA was extracted from an overnight culture using a QIAamp DNA mini kit (Qiagen), as per the manufacturer’s instructions. DNA quality was assessed using Nanodrop spectrophotometry (Thermofisher) and quantity was assessed using the Qubit 3.0 system (Thermofisher). Whole-genome sequencing was performed using Ion Torrent with 400 bp chemistry (PGM; Life Technologies) and MinION nanopore sequencing (Oxford Nanopore Technologies), as per the manufacturers' instructions. Hybrid genome assembly was performed to get the complete genome with Ion Torrent (short reads) and MinION (long reads) using Unicycler v0.4.6, as described previously [10].

Annotation was performed using the National Center for Biotechnology Information Prokaryotic Genome Annotation Pipeline (PGAP; http://www.ncbi.nlm.nih.gov/genomes/static/Pipeline.html). The downstream analysis was done using the Center for Genomic Epidemiology (CGE) server (http://www.cbs.dtu.dk/services). The resistance gene profile and plasmids were identified using ResFinder 3.0 and PlasmidFinder 1.3, respectively [11, 12]. Circular genomes of the isolates were mapped against the reference genome using CGview software [13]. Plasmid comparison was done using Gview software [14]. Additionally, integrons, prophages and insertion sequences were predicted using web tools such as phast and ISsaga [15, 16].

## Results

### Antimicrobial-susceptibility testing and resistance gene PCR

Among the total of 332 *Shigella* isolates, 31 isolates (6 *S. flexneri* and 25 *S. sonnei *) were resistant to azithromycin using the disc diffusion method as per the epidemiological cut-off value of *S. flexneri *. The azithromycin MIC for these *Shigella* isolates ranged between 2 and 16 mg l^−1^. The most common multi-drug resistant (MDR) phenotypes among *S. flexneri* and *S. sonnei* isolates in this study were AMP-SXT-CIP and SXT-CIP-AZM, respectively (Table 1). Furthermore, among the 31 resistant isolates, the *mphA* gene was only identified in one *S. sonnei* isolate (FC1428) on PCR analysis. None of the other isolates harboured the gene.

**Table 1.** Antimicrobial-resistance patterns and macrolide PCR results of the study isolates

Isolate ID	Organism	Antimicrobial-resistance pattern (DD)	Azithromycin MIC	Macrolide PCR	
				***mphA***	***ermB***
FC601	* S. flexneri* 1	AMP-SXT-CIP-AZM	2 S	−	−
FC766	* S. sonnei *	SXT-CIP-AZM	4 S	−	−
FC1084	* S. sonnei *	SXT-CIP-AZM	8 S	−	−
FC1145	* S. flexneri* 2	AMP-SXT-CIP	2 S	−	−
FC1401	* S. sonnei *	SXT-CIP-AZM	8 S	−	−
FC1433	* S. sonnei *	SXT-CIP-FIX-AZM	4 S	−	−
FC1644	* S. flexneri* 6	SXT-CIP-AZM	2 S	−	−
FC1715	* S. sonnei *	SXT-CIP-AZM	2 S	−	−
FC1879	* S. sonnei *	SXT-CIP-AZM	4 S	−	−
FC3071	* S. flexneri* 2	AMP-SXT-CIP	4 S	−	−
FC1446	* S. sonnei *	AMP-SXT-CIP-TAX-FIX-AZM	4 S	−	−
FC1429	* S. sonnei *	AMP-SXT-CIP-AZM	4 S	−	−
FC1428	* S. sonnei *	AMP-SXT-CIP-TAX-FIX-AZM	16 S	**+**	−
FC216	* S. flexneri* 2	AMP-SXT-CIP	2 S	−	−
FC1651	* S. sonnei *	SXT-CIP-AZM	2 S	−	−
FC1846	* S. sonnei *	SXT-CIP-AZM	2 S	−	−
FC1871	* S. sonnei *	SXT-AZM	8 S	−	−
FC2057	* S. sonnei *	SXT-CIP-AZM	4 S	−	−
FC1985	* S. sonnei *	AMP-SXT-CIP-AZM	8 S	−	−
FC2239	* S. sonnei *	AMP-SXT-CIP-TAX-FIX-AZM	4 S	−	−
FC1955	* S. sonnei *	SXT-CIP-AZM	4 S	−	−
FC1640	* S. flexneri* 2	AMP-SXT-CIP-TAX-FIX-AZM	8 S	−	−
FC1740	* S. sonnei *	SXT-CIP-AZM	4 S	−	−
FC1751	* S. sonnei *	SXT-CIP-AZM	4 S	−	−
FC1964	* S. sonnei *	SXT-CIP-AZM	4 S	−	−
FC1988	* S. sonnei *	SXT-CIP-AZM	12 S	−	−
FC1970	* S. sonnei *	AMP-SXT-CIP-AZM	14 S	−	−
FC2001	* S. sonnei *	SXT-CIP-AZM	16 S	−	−
FC2069	* S. sonnei *	SXT-CIP-AZM	2 S	−	−
FC794	* S. sonnei *	SXT-CIP-AZM	16 S	−	−
FC1589	* S. sonnei *	SXT-CIP-AZM	4 S	−	−

AMP, Ampicillin; AZM, azithromycin; CIP, ciprofloxacin; FIX, cefixime; SXT, trimethoprim/sulfamethoxazole; TAX, cefotaxime; R, resistant; S, susceptible; –, negative; +, positive.

DD, Disc diffusion; MIC, Minimum inhibitory concentration.

### Whole-genome sequencing

**Fig. 1.**
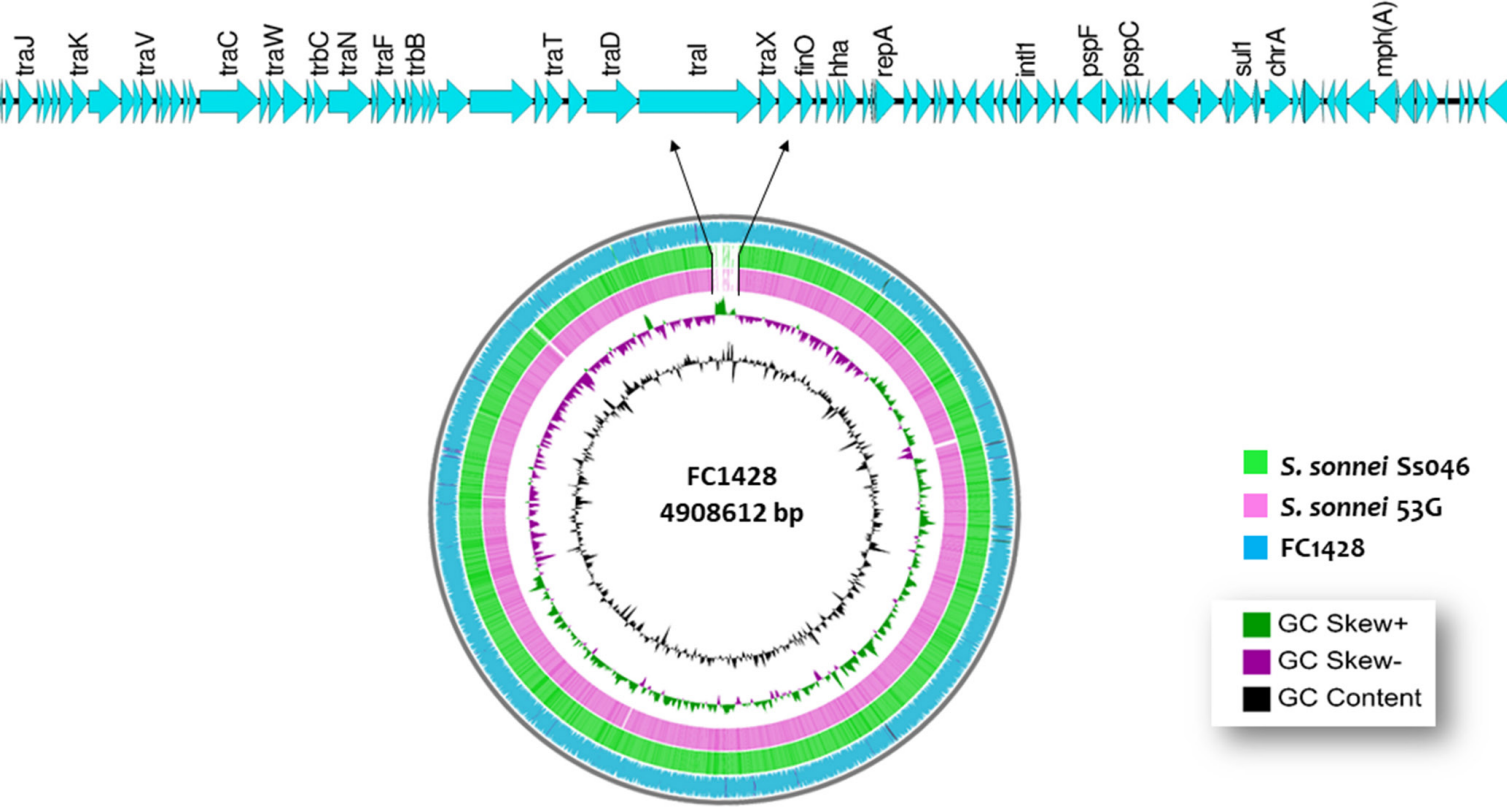
Circular representation of chromosomal integration of the IncFII plasmid carrying *mph*A in *S. sonnei *. The gap represents the integrated plasmid and the arrows indicate the genetic arrangement of the plasmid

The *S. sonnei* isolate (FC1428) that carried the *mphA* gene, and which had an azithromycin MIC value of 16 mg l^−1^, was sequenced to characterize the resistance mechanism. Sequence analysis revealed that the IncFII plasmid was integrated into the chromosome, which showed 100 % similarity to an *Escherichia coli* plasmid, pC15-1a (accession number AY458016.1) on blast analysis. The plasmid also carried the following AMR genes, *sul1*, *bla*_DHA1_, *qnrB4*, *mphA* and *tetR*, as shown in Fig. 1. In addition, the *dfrA17* gene was identified in the chromosome. The isolate also harboured the following mutations in the quinolone-resistance-determining region (QRDR): *gyrA* – S83L, D87G; and *parC* – S80I. Furthermore, class 1 and 2 integrons were identified within the plasmid. The isolate carried ten intact, nine incomplete and six questionable regions of the prophage. Also, a total of 465 IS elements was predicted to be present in the *S. sonnei* genome. The most commonly identified type was from the IS*1* family, accounting for approximately 37 % of the IS elements, followed by the IS*3*_ssgr_IS*3*, IS*4*, IS*3*_ssgr_IS*2* and IS*21*.

**Fig. 2.**
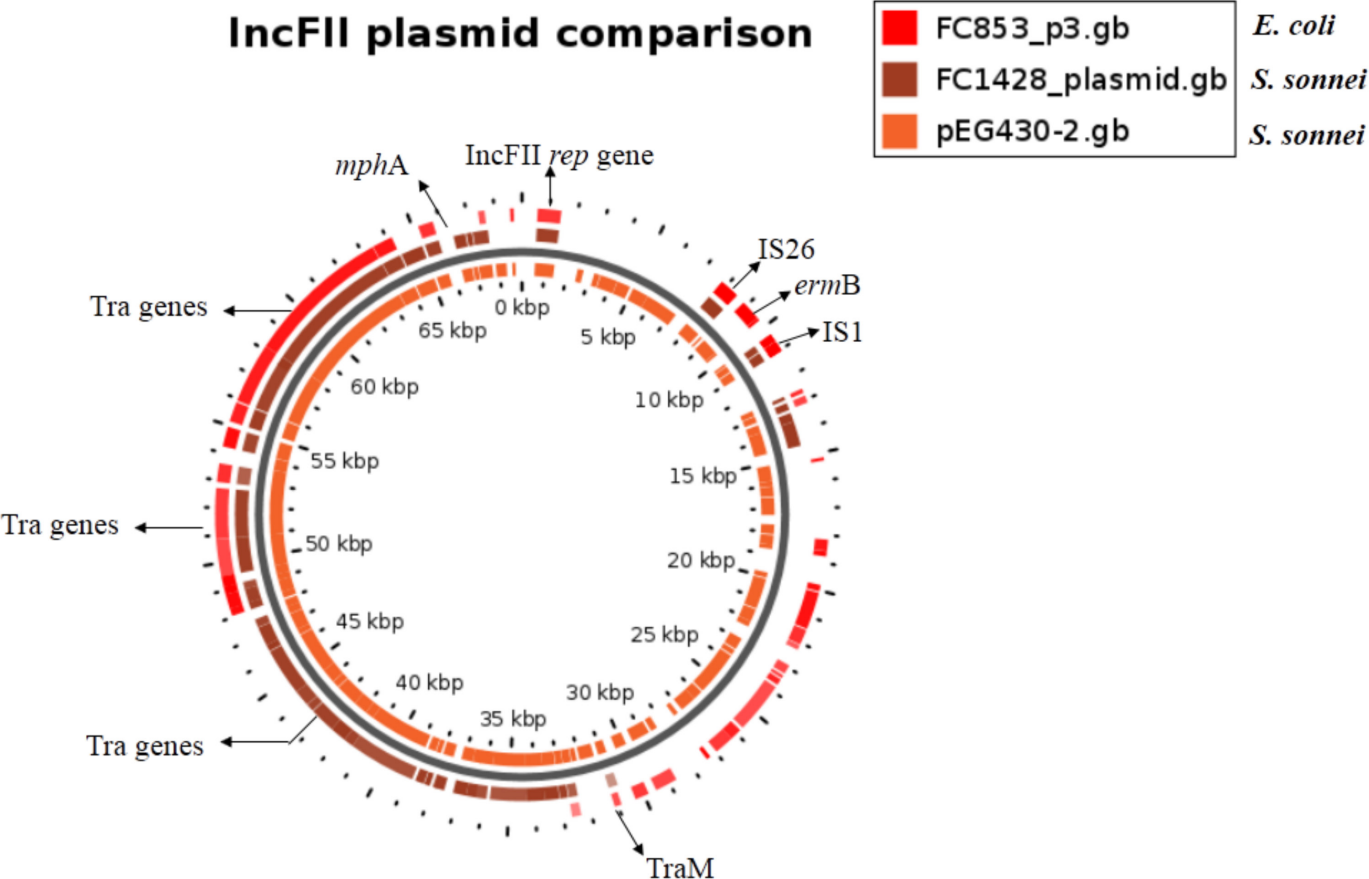
Comparison of plasmid-carried azithromycin-resistance genes using blast Atlas through the Gview server. Genes displayed are those showing similarity between the reference plasmid and the plasmid identified in this study

The plasmid region of the *S. sonnei* (FC1428) isolate was compared with the previously published azithromycin-resistance plasmids of *S. sonnei *, pEG430-2 (accession number LT174531.1), and *E. coli *, FC853 plasmid 3 (accession number CP040922) carrying the *ermC* and/or *ermB* genes. The analysis showed that the *Shigella* plasmids carried additional *tra* genes when compared to the *E. coli* plasmid, as shown in Fig. 2. The compared plasmids have the *repA* gene for the IncFII plasmid, insertion sequences such as IS*1* and IS*26*, hypothetical proteins, type II toxin–anti-toxin, pilus and *finO* genes in common. The genome sequences of the *S. sonnei* isolate has been made available in GenBank under the accession number CP041322.

## Discussion

Antibiotics are believed to reduce the duration of illness caused by *Shigella* spp. Azithromycin is often used to treat MDR, including fluroquinolone-resistant, pathogens [1]. Although azithromycin is being used worldwide for shigellosis, the lack of interpretative guidelines and only limited clinical data leads to uncertainty about the clinical outcome. Recently, the emergence of decreased susceptibility to azithromycin (DSA) has been reported in *Shigella* spp. [17, 18]. There are also reports of azithromycin-treatment failure, particularly in men who have sex with men and human immunodeficiency virus patients with shigellosis [19, 20]. This indicates that the determination of clinical breakpoints and their correlation with the clinical outcome in azithromycin-treated patients is urgently required.

In this study, the correlation of the MIC with a resistance gene showed that the MIC ranged between 2 and 16 mg l^−1^ among the study isolates, and that the *mphA* gene was only found in the *S. sonnei* isolate with a MIC of 16 mg l^−1^. Similarly, a study by Darton *et al*., showed that *S. flexneri* isolates with a MIC of 16 mg l^−1^ were positive for either *mphA* or *ermB* genes [2]. In contrast, another study by Salah *et al*. showed that the isolates with a MIC of 16 mg l^−1^ were found to be negative for all tested macrolide-resistance genes. However, both *S. flexneri* and *S. sonnei* isolates with an azithromycin MIC of ≥32 mg l^−1^ have been found to be positive for the *mphA* gene [3]. There are also reports of *E. coli* isolates with a MIC of ≥256 mg l^−1^ carrying the *mphA* gene, followed by *ermB* and *mphB* in isolates with a MIC of >1024 and 128 mg l^−1^, respectively [5].

Generally, azithromycin-resistance genes such as *mphA* and *ermB* were reported to be carried in plasmids [2]. Remarkably, in this study, the plasmid carrying the *mphA* gene was found to be integrated into the chromosome of *S. sonnei *. However, the phenomenon can be reversible if the integration is through homologous recombination, and this might be possible due to the presence of various insertion sequences and other mobile elements in *Shigella* spp. Apart from this unique finding, genome analysis revealed the presence of multiple resistance genes that were expected to be present. The mutations we observed in the QRDR are commonly reported to be associated with fluoroquinolone resistance in *Shigella* spp., as reported in previous studies [7]. The genome also contained various mobile genetic elements that are reported to play a significant role in AMR dissemination in *Shigella* spp.

Earlier, a study by Nguyen *et al*. showed that *E. coli* acts as a reservoir for macrolide-resistance genes from which resistant *Shigella* spp. might have emerged through horizontal gene transfer. The phenomenon has been previously demonstrated with *E. coli* donating *mphA* to *S. sonnei* [5]. In this study, we looked for the occurrence of a similar event among the studied isolates. We compared the IncFII plasmid profile of *Shigella* and *E. coli* carrying the macrolide-resistane gene to identify the backbone similarity. Though the analysis revealed several genes in common, the *Shigella* plasmid harboured an additional *tra* operon compared to *E. coli *, which might have been acquired due to evolution over time. The *tra* genes had always been the only genomic factors that make the plasmid conjugative.

Interestingly, Benz *et al*., in their study, showed that the plasmid transfer is mainly based on the functional *tra* (transfer) genes rather than the plasmid types [20]. Furthermore, earlier studies have shown that plasmids carrying required functional *tra* genes can spread even without antibiotic selection pressure [20]. These highlight the potential risk of plasmids with resistance genes carrying functional *tra* genes being transferred by natural conjugation.

The widespread emergence of MDR *Shigella* with changing AMR patterns has also being reported. Generally, in *Shigella* spp., acquired resistance is more common. β-Lactam resistance is mainly due to the presence of OXA-type β-lactamases, followed by TEM and CTX-M. Trimethoprim/sulfamethoxazole resistance is encoded by *dhfr1A* and *sul* genes. Quinolone resistance involves the accumulation of mutations in QRDR and plasmid-mediated quinolone resistance (PMQR) genes. Furthermore, resistance to tetracycline, chloramphenicol and streptomycin has been shown to be due to the presence of *tetA*/*B*, *catA1* and of either *strA*/*B* or *aadA1* genes or both [7]. These show the species ability in acquiring AMR determinants.

### Conclusion

To conclude, the novel finding of an integrated plasmid in this study indicates the potential risk of *Shigella* isolates becoming resistant to azithromycin in the future. Characterization of the functional *tra* genes is important to track the potential dissemination of macrolide resistance. The study also highlights the significance of the hybrid assembly approach in the complete genome analysis. These findings suggest that it is imperative to monitor *Shigella* susceptibility and to study the resistance mechanism of *Shigella* against azithromycin, considering the limited treatment choices for shigellosis.
